# Hass Avocado Bioactive Compounds Attenuating Oxidative Stress and Inflammation in Ischemia–reperfusion Injury: An Integrative Review

**DOI:** 10.1007/s11130-026-01482-4

**Published:** 2026-05-08

**Authors:** Rebeca Escutia-Gutiérrez, Walter Ángel Trujillo-Rangel, Leonel García-Benavides, Marco Pérez-Cisneros, Adalberto Zamudio-Ojeda, Ana Sandoval-Rodríguez, Juan Armendáriz-Borunda, Maykel González-Torres, Santiago José Guevara-Martínez

**Affiliations:** 1 Instituto de Biología Molecular en Medicina y Terapia Génica, Departamento de Biología Molecular y Genómica, Centro Universitario de Ciencias de la Salud, Universidad de Guadalajara, 44340 Guadalajara, Jalisco, México; 2 Departamento de Ciencias Biomédicas, Centro Universitario de Tonalá, Universidad de Guadalajara, Tonalá, 45425 Jalisco México; 3 Departamento de Aparatos y Sistemas II, Ciencias de la Salud, Universidad Autónoma de Guadalajara, Av. Patria 1201, Lomas del Valle, Zapopan, 45129 Jalisco Mexico; 4 Departamento de Electrofotónica, Centro Universitario de Ciencias Exactas e Ingenierías, Universidad de Guadalajara, Guadalajara, Jalisco, 44430 México; 5 Departamento de Física, Centro Universitario de Ciencias Exactas e Ingenierías, Universidad de Guadalajara, Guadalajara, Jalisco, 44430 México; 6 Tecnológico de Monterrey, Escuela de Medicina y Ciencias de la Salud, Zapopan, 45138 Jalisco México; 7 Departamento de Ciencias Químicas, Facultad de Estudios Superiores Cuautitlán, Universidad Nacional Autónoma de México, Cuautitlán Izcalli, Edo. de México, 54740 México; 8 Departamento de Farmacobiología, Centro Universitario de Ciencias Exactas e Ingenierías, Universidad de Guadalajara, Guadalajara, Jalisco, 44430 México

**Keywords:** Avocado, Inflammation, Ischemia-reperfusion injury, Antioxidant activity, Oxidative stress

## Abstract

Ischemia–reperfusion injury (IRI) is a complex and clinically significant pathophysiological process that occurs when blood flow is restored to an organ following ischemia. While reperfusion is essential for tissue survival, it paradoxically exacerbates damage through mechanisms such as oxidative stress, inflammation, mitochondrial dysfunction, and endothelial injury. This phenomenon contributes significantly to morbidity and mortality in various clinical settings, including organ transplantation, myocardial infarction, stroke, and liver and kidney injuries. Despite increasing knowledge of the molecular pathways involved, effective treatment options remain limited. Recent scientific advances have highlighted the potential of natural bioactive compounds to reduce oxidative and inflammatory responses during IRI. Hass avocado (*Persea americana* Mill.) has garnered considerable attention as a rich source of phytochemicals with strong antioxidant and anti-inflammatory properties, including carotenoids, tocopherols, polyphenols, and monounsaturated fatty acids (MUFAs). These compounds may act synergistically to neutralize reactive oxygen species, inhibit proinflammatory mediators, and modulate key cellular pathways involved in IRI. This comprehensive review explores the current evidence regarding the protective effects of Hass avocado bioactive compounds against IRI. We examined their molecular mechanisms of action and discussed the therapeutic implications of incorporating these natural agents into clinical practice. To our knowledge, this is the first review specifically addressing the role of Hass avocado phytochemicals in reducing oxidative damage and inflammation in IRI, offering a novel perspective for future research and clinical application.

**Graphical Abstract:**

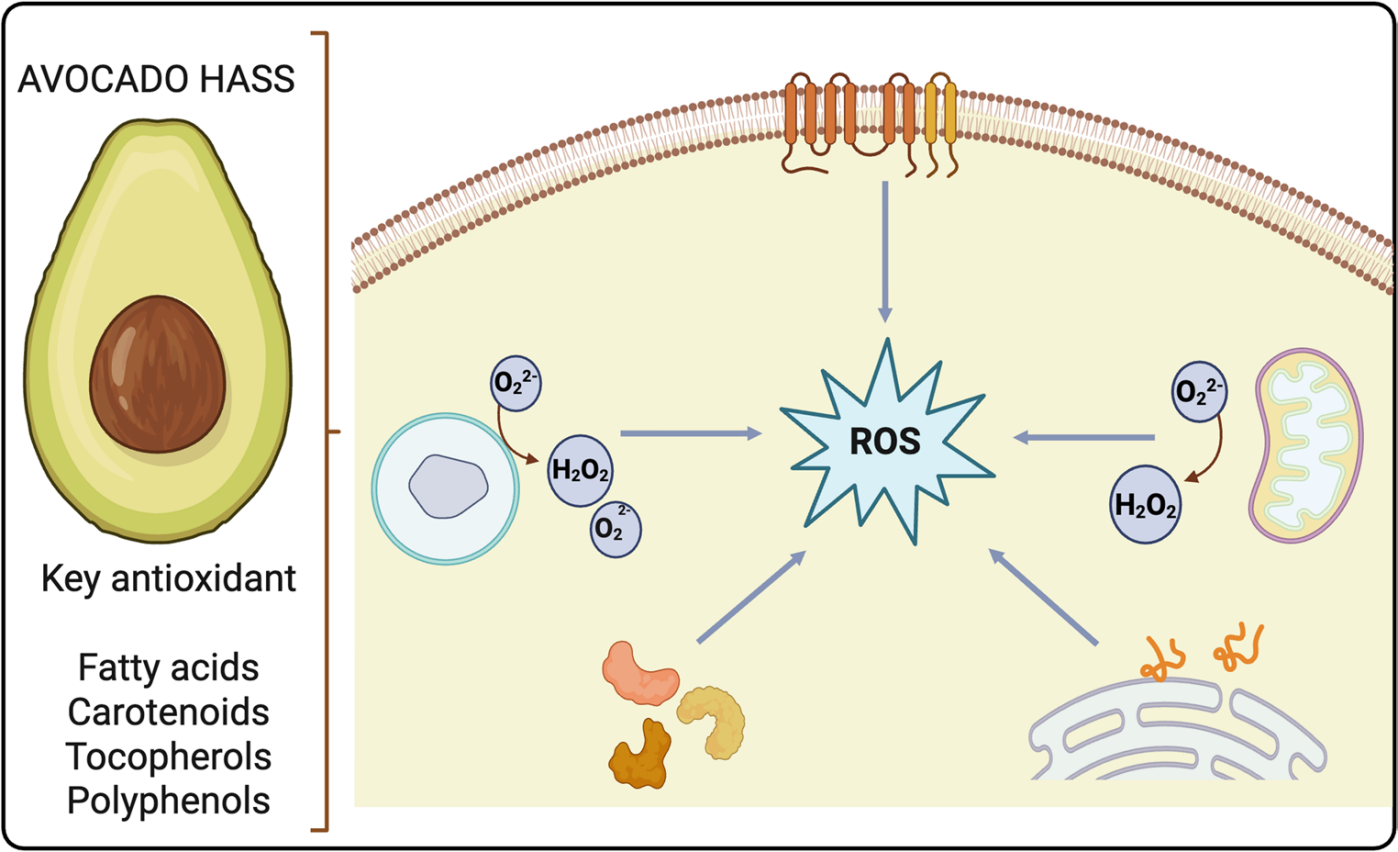

**Supplementary Information:**

The online version contains supplementary material available at 10.1007/s11130-026-01482-4.

## Introduction

Ischemia-reperfusion injury (IRI) presents a significant challenge in modern medicine and manifests as various clinical conditions, including organ transplantation, myocardial infarction, and stroke. This pathological condition is characterized by an initial restriction of blood flow to an organ, followed by restoration of perfusion and oxygenation, which paradoxically induces further damage through mechanisms such as oxidative stress and inflammation [1]. Therefore, it is crucial to develop effective strategies to alleviate these effects, considering the extensive influence of IRI on patient outcomes and healthcare systems [2].

Previous studies have comprehensively reviewed the molecular pathways involved in ischemia-reperfusion injury and have discussed potential therapeutic interventions [3]. Clinical implications and mechanistic insights of ischemia-reperfusion injury, particularly in the liver, have been extensively reviewed [4]. A bibliometric analysis has provided insights into the research trends and developments in myocardial ischemia-reperfusion injury over the past decade [5]. However, to the best of our knowledge, no comprehensive review has examined the bioactive compounds of Hass avocados and their role in attenuating oxidative damage and inflammation during ischemia–reperfusion injury.

Recent advancements in our understanding of natural bioactive compounds present promising avenues for the development of novel therapeutic agents. Notably, Hass avocados (*Persea americana* Mill.) have emerged as a significant source of these compounds, which exhibit antioxidative and anti-inflammatory effects. This review comprehensively analyzes the bioactive components of Hass avocados, focusing on their roles in attenuating oxidative damage and inflammation during intestinal ischemia–reperfusion (IRI). Through this analysis, we explore the molecular mechanisms by which these compounds influence cellular processes and discuss their implications for clinical practice and future research.

## Ischemia‒Reperfusion Injury

Ischemia reperfusion injury (IRI) refers to cellular and tissue damage resulting from the restoration of blood flow to previously hypoperfused, hypoxic tissues. Following reperfusion of tissues subjected to arterial occlusion, a lesion is formed that accelerates the development of necrosis. The impact of this pathological phenomenon on health systems is substantial, necessitating the implementation of strategies for epidemiological control and prevention [6]. IRI occurs in a variety of pathologies and interventions and is invariably observed in conditions such as cardiac, thoracic, peripheral vascular, and major vascular surgery, or solid organ transplantation. Despite the high prevalence of ischemia, effective treatment and prevention of injury to resolve the induced damage [7]. Pharmacological preconditioning offers one of the most efficient therapeutic alternatives, preparing tissues for potential ischemic events. Although there is a predominant emphasis on medication treatments, it is widely recognized that effective control of oxidative stress and the inflammatory response is crucial for proper alleviation of ischemia reperfusion injury [8].

## Injury Process in Ischemia‒Reperfusion

Multiple aspects of IRI are primarily associated with oxidative stress, inflammation, calcium overload, energy metabolism disorders, pyroptosis, and ferroptosis, all of which are determinants of tissue injury. Ischemia interrupts arterial blood flow to tissues or organs, resulting in an imbalance of metabolic substrates and tissue hypoxia [9]. Subsequently, the re-establishment of blood flow and reoxygenation are linked to the exacerbation of local tissue injury and severe local and systemic inflammatory responses. IRI induces the generation of reactive oxygen species (ROS) due to oxidative stress, which is a crucial event in this type of injury [10]. ROS accumulation exacerbates tissue damage and activates oxidative processes that affect lipids, proteins, and nucleic acids. Reperfusion triggers a cascade of events that aggravate potentially harmful local inflammatory responses [11].

Pyroptosis is distinguished from apoptosis and necroptosis by the formation of cell membrane pores. Ferroptosis, an alternative form of cell death to apoptosis, is characterized by elevated levels of iron-dependent lipid hydroperoxide accumulation and is not influenced by substances associated with other recognized cell death pathways. Consequently, ischemic reperfusion injury (IRI) is distinct from other cell death mechanisms in terms of morphology, biochemistry, and genetics. It has several clinical implications, including its role in ischemia. Furthermore, owing to its close association with various oxidative components in this condition, it represents a critical oxidative pathway that can influence IRI [12].

## The Molecular Mechanism Involved in Oxidative Damage

Hypoxia-inducible factors (HIFs) are oxygen-regulated transcription factors essential for sensing and adapting to low oxygen levels (hypoxia). These factors regulate the expression of numerous genes in response to hypoxia. Specifically, hypoxia-inducible factor 1α (HIF-1α) expression and mitochondrial reactive oxygen species (ROS) generation increase during ischemic oxidative stress [13]. Under hypoxic conditions, HIF-1α stabilization occurs, which is linked to elevated ROS levels generated by complex III in the mitochondria. This stabilization is associated with the oxidative inactivation of nonheme iron at the catalytic site of the prolyl hydroxylase (PHD) enzyme, which normally degrades HIF-1α under normoxic conditions [14].

HIF-1α accumulation exerts distinct effects contingent on the cell type. In the brain, HIF-1α signaling is associated with neuroprotection, promotion of metabolic adaptation, and neuronal survival. However, excessive activation of cerebral endothelial cells may lead to blood–brain barrier disruption during reperfusion [15]. In the context of myocardial ischemia-reperfusion, HIF-1α activation facilitates cardiomyocyte survival and angiogenic responses partially through the induction of vascular endothelial growth factor (VEGF). However, in hepatic and renal ischemia–reperfusion, HIF-1α signaling plays a role in metabolic reprogramming and cytoprotective responses; however, prolonged activation may also intensify inflammatory and fibrotic pathways [13]. Reactive oxygen species (ROS) generated during ischemia and early reperfusion act as signaling molecules, activating redox-sensitive transcription factors, such as nuclear factor kappa B (NF-κB) and nuclear factor erythroid 2-related factor 2 (NRF2). NF-κB activation, triggered by ROS-mediated degradation of its inhibitor IκB, promotes the transcription of genes involved in inflammation and cell survival and is particularly relevant in renal and hepatic ischemia-reperfusion injury, where inflammatory amplification contributes to tissue damage. In contrast, NRF2 activation induces antioxidant and cytoprotective enzymes, representing a key adaptive response in multiple organs, including the heart, brain, liver, and kidney [16].

This facilitates NF-κB translocation to the nucleus and the subsequent activation of genes involved in inflammation and cell survival [17]. Furthermore, ROS-mediated activation of the MAPK pathway enhances VEGF expression, and VEGF, along with its receptors (VEGFR1 and VEGFR2), promotes angiogenesis, cell survival, and adaptation to hypoxia via MAPK signaling and direct activation of HIF-1α. This mechanism is particularly relevant in cardiac and cerebral ischemia-reperfusion models, in which vascular remodeling and perfusion recovery are critical for tissue repair [18].

## Oxidative Stress Mechanisms

During ischemia, oxygen and nutrient supply to the affected organ or tissue decreases, leading to the accumulation of metabolic waste and cellular damage. Following reperfusion, there is a rapid influx of oxygen and nutrients, resulting in the production of ROS and nitrosative species that can cause further cellular damage [19]. This section discusses the key mechanisms underlying oxidative stress and the functions of antioxidant enzymes in intestinal ischemia–reperfusion injury.

**Fig. 1:**
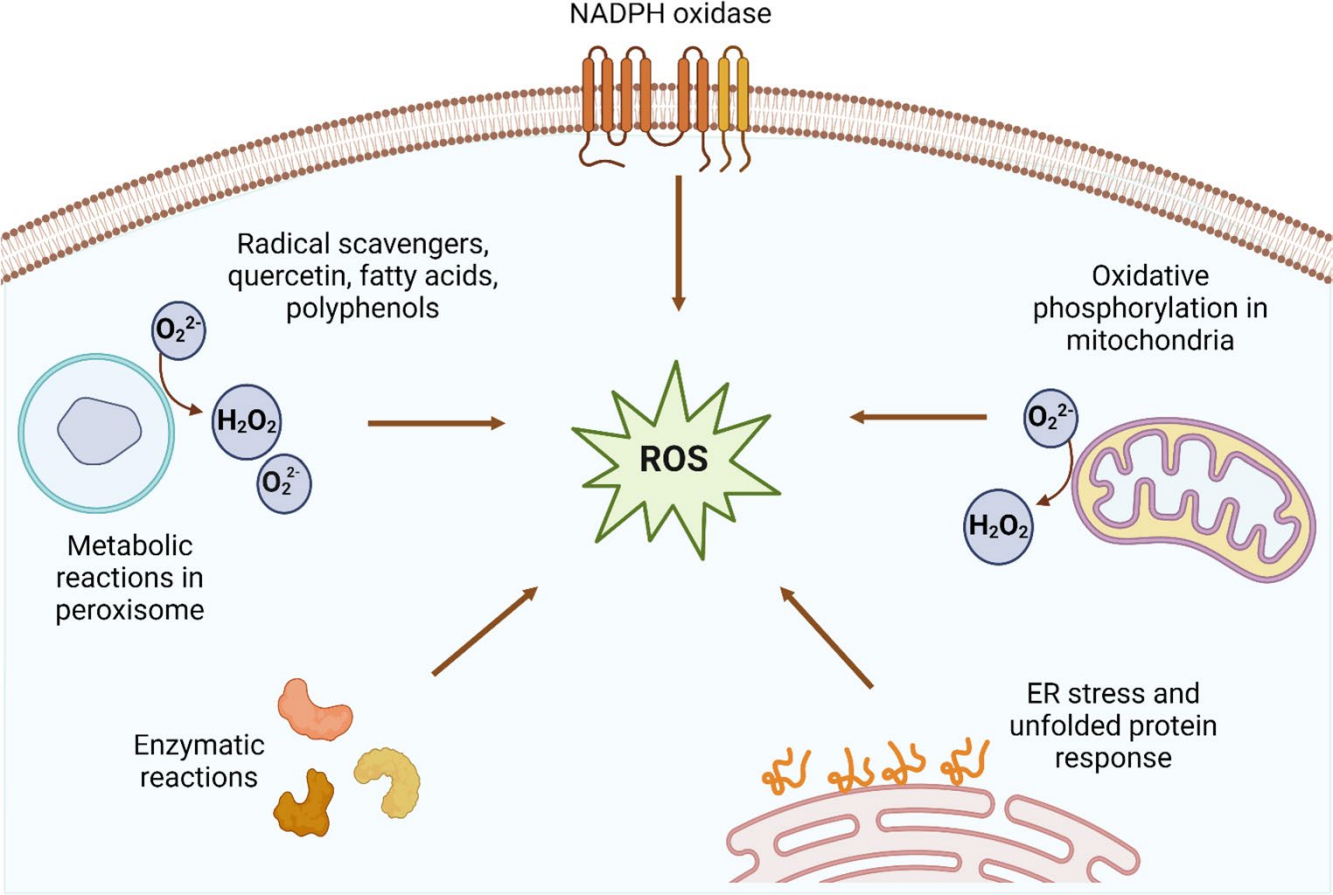
Main cellular and molecular events underlying oxidative stress during ischemia–reperfusion injury

ROS and RNS production following IRI damage results from the complex interplay of multiple cellular pathways. During ischemia, mitochondrial function and adenosine triphosphate (ATP) production decrease, leading to an increase in electron leakage and the production of superoxide anions (O_2_^−^). Upon reperfusion, the influx of oxygen and nutrients can lead to the production of additional ROS, including hydrogen peroxide (H_2_O_2_) and hydroxyl radicals (OH^−^). These ROS can cause oxidative damage to cellular structures, such as lipids, proteins, and deoxyribonucleic acid (DNA), leading to cell death and tissue injury (Fig. 1) [20].

Ischemia-reperfusion injury results from the simultaneous activation of multiple cellular pathways, leading to excessive reactive oxygen species (ROS) production and subsequent oxidative damage. Major sources of ROS include nicotinamide adenine dinucleotide phosphate (NADPH) oxidase in endothelial and inflammatory cells, enhanced peroxisomal metabolism generating hydrogen peroxide, and mitochondrial electron transport chain dysfunction, particularly electron leakage at complexes I and III during oxygen reintroduction. Additional contributions come from enzymes such as xanthine oxidase and cyclooxygenases, as well as endoplasmic reticulum stress and unfolded protein response activation, which further exacerbate oxidative stress through disrupted calcium homeostasis and oxidative signaling pathways (Fig. 1) [21].

## Roles of Antioxidant Enzymes

Antioxidant enzymes play a crucial role in preventing oxidative damage during ischemia–reperfusion injury. Superoxide dismutase (SOD) is an enzyme that catalyzes the conversion of superoxide anions to hydrogen peroxide and oxygen, thereby preventing the formation of more reactive oxygen species. Catalase (CAT) and glutathione peroxidase (GPH) catalyze the breakdown of hydrogen peroxide and lipid hydroperoxides, respectively, thereby preventing the formation of additional reactive oxygen species. In addition to these enzymes, tripeptide glutathione serves as an antioxidant and free radical scavenger, mitigating oxidative damage (Fig. 1) [22].

Reactive oxygen species and reactive nitrogen species function as both damaging agents and signaling molecules during ischemia-reperfusion injury. Targeting these species with antioxidant compounds can interrupt inflammatory cascades and mitigate cellular damage. Myeloperoxidase (MPO), an enzyme released by activated neutrophils during inflammation, generates highly reactive oxidants, including hypochlorous acid. Inhibition of MPO activity reduces oxidative stress and subsequent inflammatory damage. Microsomal triglyceride transfer protein (MTTP) regulates lipid metabolism and can influence inflammatory responses by modulating lipid profiles and oxidative stress. Endothelial nitric oxide synthase (eNOS) produces nitric oxide, which promotes vascular health under normal conditions. However, during oxidative stress, eNOS can become uncoupled, producing superoxide instead of nitric oxide, which contributes to endothelial dysfunction. Peroxisome proliferator-activated receptor gamma (PPAR-*γ*) functions as an anti-inflammatory transcription factor, suppressing inflammatory gene expression and promoting the resolution of inflammation. Cell-cell adhesion molecules (CAMs) mediate leukocyte recruitment to inflamed tissues. Reducing CAM expression through antioxidant interventions decreases inflammatory cell infiltration and subsequent tissue damage during ischemia-reperfusion [23].

## Modulators of Enzyme Activity

Enzyme activity is modulated by factors including pH, temperature, and molecules such as inhibitors or activators. These modulators can regulate enzyme activity and potentially treat diseases resulting from enzyme dysregulation. For example, cyclooxygenase (COX) inhibitors are utilized as anti-inflammatory drugs. Conversely, activators of the enzyme glucokinase are being investigated as potential therapies for diseases characterized by ATP generation, the cell’s primary energy currency. Mitochondrial respiratory chain blockers inhibit this process, thereby reducing ROS production. These compounds possess therapeutic potential in a range of disorders, including cancer, neurodegenerative diseases, and IRI damage [24].

Oxidative stress in plant-derived foods under ischemia–reperfusion–like conditions results from the activation of multiple interconnected cellular pathways that enhance reactive oxygen species generation. Major ROS sources include NADPH oxidase activity, mitochondrial electron leakage during oxidative phosphorylation, peroxisomal metabolic reactions, oxidative enzymes, and endoplasmic reticulum stress associated with UPR. These processes accelerate the oxidative damage to lipids, proteins, and bioactive compounds, ultimately compromising the nutritional quality and functional properties of plant foods. Avocado-derived bioactive compounds, along with other antioxidants such as quercetin, fatty acids, and polyphenols, mitigate oxidative stress by modulating mitochondrial function and NADPH oxidase–related pathways, thereby reducing excessive ROS production and oxidative damage. NADPH, nicotinamide adenine dinucleotide phosphate; ROS, reactive oxygen species; UPR, unfolded protein response.

Oxidative stress–induced activation of NF-*κ*B promotes the transcription of pro-inflammatory mediators, including cytokines, chemokines, and adhesion molecules. The inhibition of this pathway by antioxidant compounds can effectively reduce inflammatory responses. In contrast, Nrf2 acts as a central regulator of antioxidant and cytoprotective defenses, inducing the expression of detoxifying and antioxidant enzymes. Therapeutic modulation that suppresses NF-*κ*B signaling while enhancing Nrf2 activation represents a promising strategy for mitigating oxidative stress and inflammation during ischemia–reperfusion. By limiting oxidative damage to lipids, proteins, and nucleic acids, avocado bioactives may indirectly reduce the activation of redox-sensitive inflammatory pathways, including NF-*κ*B signaling. NF-*k*B: nuclear factor kappa B; NRF2: nuclear factor erythroid 2–related factor 2; ROS: reactive oxygen species; RNS: reactive nitrogen species; MPO: myeloperoxidase; MTTP: microsomal triglyceride transfer protein; eNOS: endothelial nitric oxide synthase; PPAR-*γ*: peroxisome proliferator-activated receptor-gamma; CAMs: cell‒cell adhesion molecules.

## Pharmacological Targets That Modulate Oxidative Damage by Ischemia‒Reperfusion

A thorough evaluation of potential therapeutic targets functioning as receptors for antioxidant drugs is necessary to advance research toward more promising therapeutic alternatives. Specific triggers, particularly those involving cytosolic calcium overload, that induce the production of reactive species within mitochondria play a crucial role in cellular processes. In ischemia-reperfusion injury, cells experience a phase of diminished blood flow and oxygen supply during ischemia, a frequent cause of tissue damage. For example, myocardial infarction occurs when coronary blood flow is blocked, resulting in ischemia within the heart muscle. Following reperfusion–the reestablishment of blood flow–a sudden influx of oxygen paradoxically heightens oxidative stress and contributes to tissue damage. This phenomenon is evident in conditions such as strokes and organ transplantation [23].

Cytosolic calcium overload and excitotoxicity exemplify the consequences of excessive glutamate receptor activation, resulting in increased cytosolic calcium influx. Excessive glutamate release occurs in conditions such as ischemic stroke, leading to sustained calcium influx, which contributes to neuronal injury and cell death. Furthermore, dysfunction of voltage-gated calcium channels, frequently observed in neurodegenerative diseases such as Alzheimer’s disease, can disrupt calcium homeostasis and promote cellular stress. Mitochondrial dysfunction, characterized by compromised electron transport chain (ETC) activity within mitochondria, is a factor identified in neurodegenerative diseases, such as Parkinson’s disease. Mitochondrial damage in dopaminergic neurons can compromise the ETC, increasing ROS production and contributing to cell death. The opening of the mitochondrial permeability transition pore (mPTP) is implicated in conditions such as ischemic stroke, leading to calcium release and contributing to neuronal injury [25]. Glutathione reduction is a significant factor in initiating ferroptosis, a type of controlled cell death. In conditions such as acute kidney injury, oxidative stress leads to a decrease in cellular glutathione levels, rendering cells more vulnerable to lipid peroxidation and ferroptosis. Inhibition of glutathione peroxidase 4 (GPX4) represents another trigger, and its dysregulation has been implicated in conditions such as cancer, where GPX4 inhibition promotes lipid peroxidation and cell death [26]. Dysfunction of voltage-gated calcium channels is a cause of cardiovascular diseases, such as arrhythmias. Genetic mutations or pathological conditions affecting these channels can disrupt calcium homeostasis in cardiac cells, leading to abnormal electrical activity and arrhythmias that may be life-threatening. Targeting calcium channel dysfunction represents potential therapeutic avenues for managing cardiovascular disorders associated with irregular calcium handling [27].

**Fig. 2:**
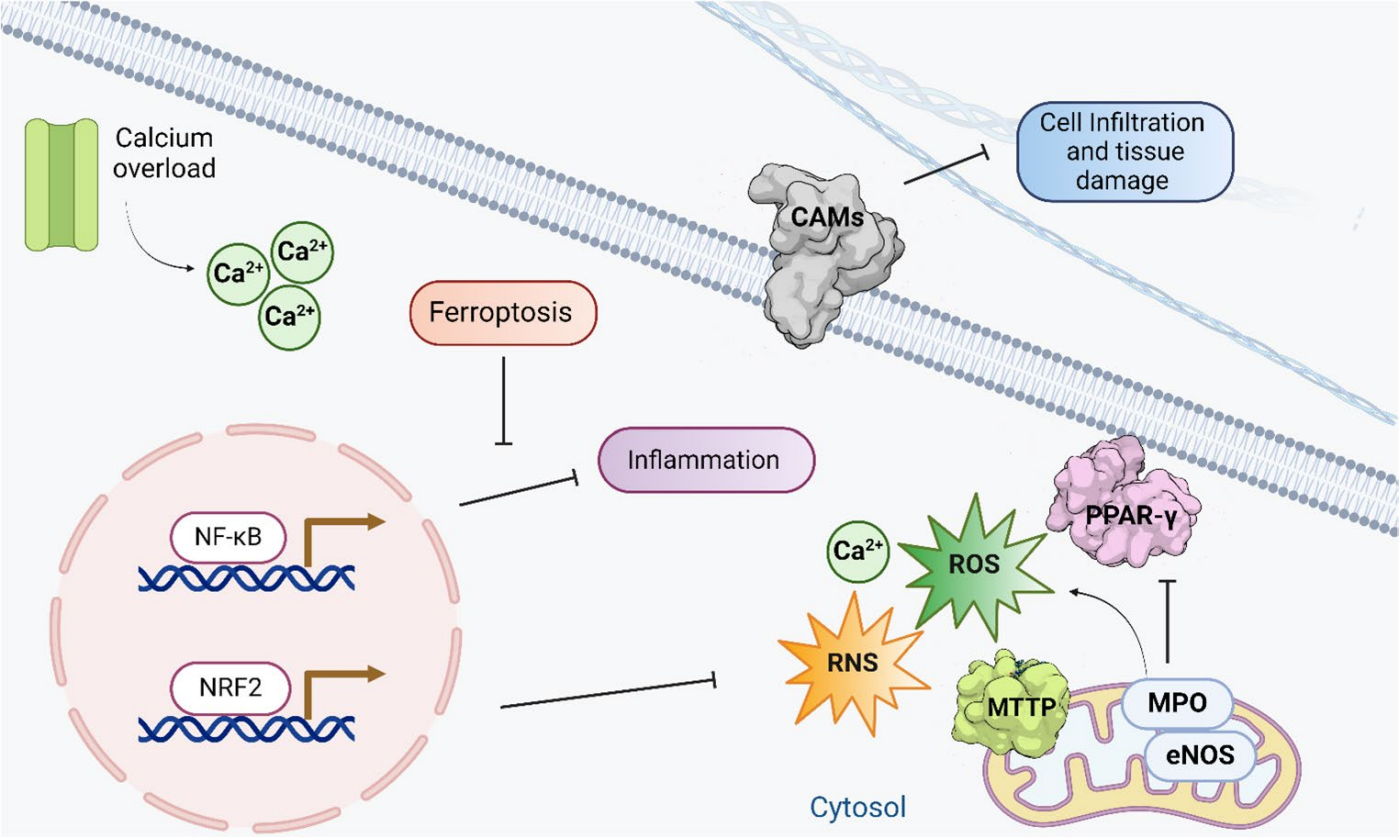
Principal oxidative targets involved in the modulation of inflammatory responses during ischemia–reperfusion injury

Antioxidant therapy mitigates oxidative stress in diverse experimental models. Generally, established strategies, such as antioxidant preconditioning using, N-acetyl cysteine, erythropoietin, and the regulation of angiotensin receptors, have yielded partially effective outcomes because the nonspecific nature of ROS modulation, which interferes with cell signaling pathways [8, 28]. Consequently, alternative approaches have emerged, including the activation of the Nrf2 pathway using compounds such as fumaric acid derivatives, which have demonstrated antioxidant activity (Fig. 2). Another noteworthy strategy involves the use of ROS-generating enzymes, such as nitric oxide synthase (NOS) and myeloperoxidase (MPO), which enable a more targeted response to modulate pathological conditions [29]. However, the most promising approach involves targeting enzyme activities, mainly through drugs with the potential to reverse endothelial nitric oxide synthase (eNOS) activity in pathologies associated with oxidative stress (Fig. 2) [30, 31].

## Anti-Inflammatory Targets in Ischemia‒Reperfusion

**Fig. 3:**
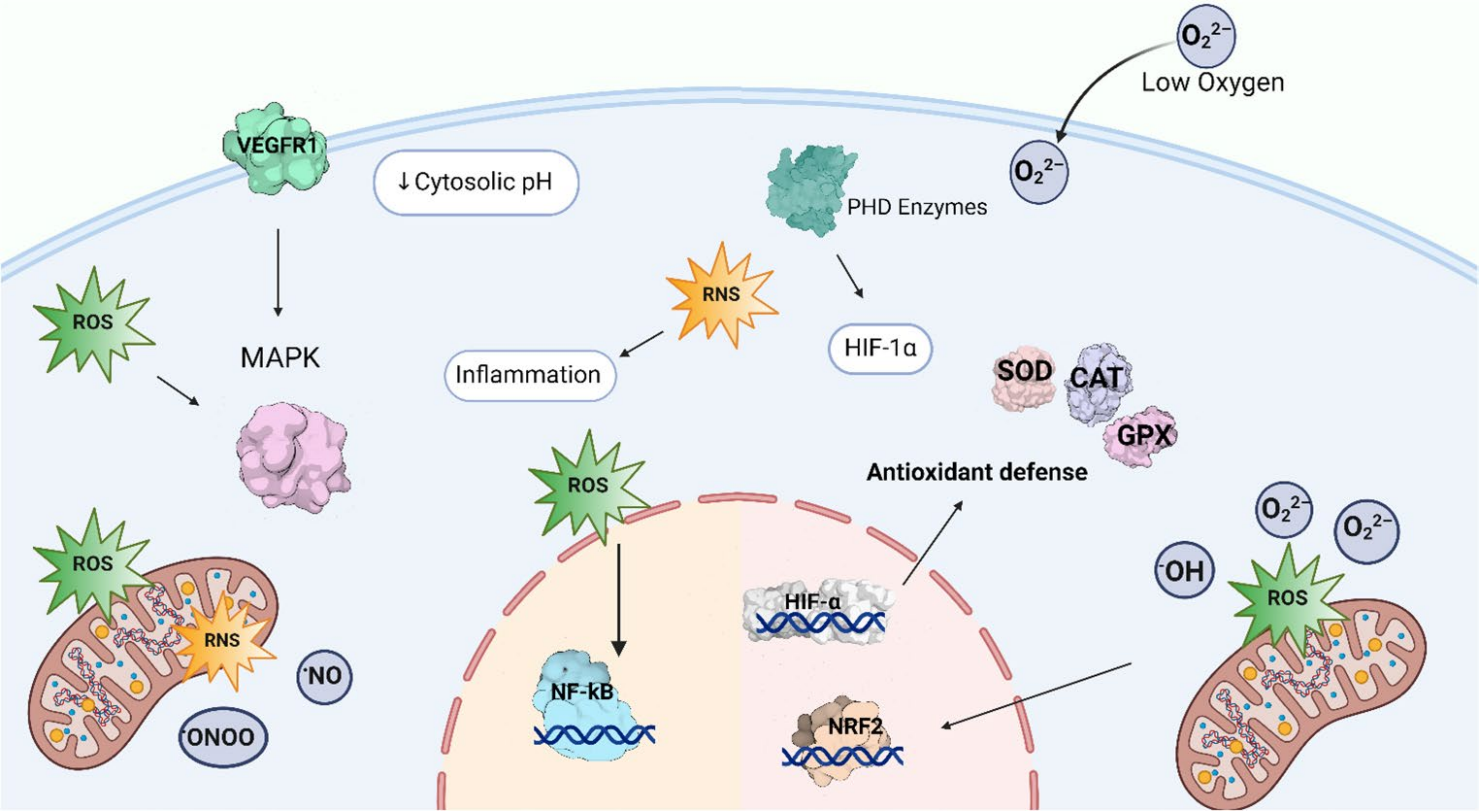
Major molecular antioxidant targets implicated in ischemia–reperfusion injury

Ischemia-reperfusion injury can lead to tissue damage and dysfunction via activation of various inflammatory pathways. This section discusses the roles of chemokines and cytokines, cell adhesion molecules, and interventions to inhibit NF-κB and Toll-like receptors in the inflammatory response following ischemia-reperfusion injury [32]. Chemokines and cytokines are small proteins produced by various cell types in response to tissue injury and inflammatory stimuli. In ischemia-reperfusion injury, chemokines and cytokines recruit immune cells to the injury site and activate them to produce a cascade of inflammatory mediators. Targeting chemokines and cytokines, including, CXCL8, CCL2, TNF-alpha, IL-1, and IL-6, has been proposed as a potential therapeutic approach for reducing inflammation and improving tissue function following ischemia-reperfusion injury [33]. Cell adhesion molecules (CAMs) are proteins expressed on the surface of various cells and involved in cell–‒cell interactions. In ischemia-reperfusion injury, CAMs mediate the adhesion of immune cells to the endothelium of blood vessels, facilitating their migration to the site of injury. Targeting CAMs is an attractive therapeutic approach for reducing immune cell infiltration and tissue damage following ischemia-reperfusion injury (Fig. 3) [34].

Hypoxia-inducible factor-1α coordinates cellular adaptation to ischemia by inducing genes associated with angiogenesis, metabolic reprogramming, and cell survival. Antioxidant defense is primarily mediated by SOD, CAT, and GPx which collectively detoxify reactive oxygen species and limit oxidative damage. Reactive oxygen and nitrogen species act as signaling molecules and mediators of cellular injury. VEGFR1 regulates angiogenic and vascular responses, Nrf2 promotes antioxidant gene expression, and NF-κB drives inflammatory signaling. Balanced modulation of these pathways is critical for limiting tissue damage during ischemia–reperfusion.

Avocado-derived compounds have been associated with the activation of endogenous antioxidant defense systems, particularly through the modulation of the Nrf2/ARE pathway and the increased activity of antioxidant enzymes, such as SOD, CAT, and GPx.

HIF-1a: Hypoxia inducible factor 1 subunit alpha SOD: superoxide dismutase; CAT: catalase; GPx: glutathione peroxidase; ROS: reactive oxygen species; RNS: reactive nitrogen species; VEGFR1: vascular endothelial growth factor receptor-1; NRF2: nuclear factor erythroid 2–related factor 2; NF-kB: nuclear factor kappa B.

## *Persea Americana* Mill cv. Hass: A Superfood

Avocado Hass is commercially appreciated and farmed in humid and Mediterranean climates worldwide. Owing to its nutritional properties, avocado is one of the major fruit crops produced commercially worldwide. Mexico is the leading producer of avocados, and the Hass variety is a medium-sized fruit characterized by a pleasant, creamy, and smooth texture. One-half of an avocado contains dietary fiber (4.6 g), total sugar (0.2 g), sodium (5.5 mg), potassium (345 mg), and magnesium (19.5 mg); some vitamins, such as vitamin A (5.0 µg RAE), vitamin C (6.0 mg), vitamin E (1.3 mg), vitamin K1 (14 µg), folate (60 mg), vitamin B-6 (0.2 mg), niacin (1.3 mg), pantothenic acid (1.0 mg), riboflavin (0.1 mg), choline (10 mg), lutein/zeaxanthin (185 µg), cryptoxanthin (18.5 µg), phytosterols (57 mg), and a high content of monounsaturated fatty acids (6.7 g) [35]; and various nutrients and phytochemicals that are individually associated with cardiovascular benefits. The avocado also contains approximately 15% monounsaturated fatty acid (MUFA)-rich oil, carbohydrates, proteins, seven-carbon sugars, and phytochemicals such as carotenoids, polyphenolic and phenolic antioxidants, phytostanols, and phytosterols, all of which are correlated with numerous potential health benefits. The constituents of the plant exhibit antifungal, anti-inflammatory, and antioxidant properties [36]. The nutritional and phytochemical compositions of Hass avocados are summarized in Table 1.

**Table 1:** Nutritional composition of Hass avocado (*Persea americana Mill.*) at varying serving amounts (USDA, 2018)

Compound	A	B	C	D
Water (g)	36.6	73.2	110	147
Energy (kcal)	80	160	240	322
Energy (KJ)	335	670	1000	1350
Protein (g)	1	2	3	4.02
Total lipid (fat) (g)	7.35	14.7	22	29.5
Ash (g)	0.79	1.58	2.37	3.18
Carbohydrate, by difference (g)	4.26	8.53	12.8	17.1
Fiber, total dietary (g)	3.35	6.7	10	13.5
Sugars, total including NLEA (g)	0.33	0.66	0.99	1.33
Sucrose (g)	0.03	0.06	0.09	0.121
Glucose (dextrose) (g)	0.185	0.37	0.555	0.744
Fructose (g)	0.06	0.12	0.18	0.241
Starch (g)	0.055	0.11	0.165	0.221
Minerals				
Calcium, Ca (mg)	6	12	18	24.1
Iron, Fe (mg)	0.275	0.55	0.825	1.1
Magnesium, Mg (mg)	14.5	29	43.5	58.3
Phosphorus, P (mg)	26	52	78	105
Potassium, K (mg)	242	485	728	975
Sodium, Na (mg)	3.5	7	10.5	14.1
Zinc, Zn (mg)	0.32	0.64	0.96	1.29
Copper, Cu (mg)	0.095	0.19	0.285	0.382
Manganese, Mn (mg)	0.071	0.142	0.213	0.285
Selenium, Se (µg)	0–2	0.4	0.6	0.804
Fluoride, F (µg)	3.5	7	10.5	14.1
Vitamins				
Vitamin C, total ascorbic acid (mg)	5	10	15	20.1
Thiamin (mg)	0.034	0.067	0.101	0.135
Riboflavin (mg)	0.065	0.13	0.195	0.261
Niacin (mg)	0.87	1.74	122	3.5
Vitamin B6 (mg)	0.129	0.257	0.386	0.517
Folate, total (µg)	40.5	81	122	163
Vitamin A, RAE (µg)	3.5	7	10.5	14.10
Vitamin E (alpha-tocopherol) (mg)	1.04	2.07	3.1	4.16
Vitamin K (phylloquinone) (µg)	10.5	21	31.5	42.2
Fatty acids				
Fatty acids, total saturated (g)	1.06	2.13	3.2	4.28
SFA 16:0 (g)	1.04	2.08	3.12	4.18
SFA 18:0 (g)	0.025	0.049	0.074	0.098
Fatty acids, total monounsaturated (g)	4.9	9.8	14.7	19.7
MUFA 16:1 (g)	0.349	0.698	1.05	1.4
MUFA 17:1 (g)	0.005	0.01	0.015	0.02
MUFA 18:1 (g)	4.54	9.07	13.6	18.2
Fatty acids, total polyunsaturated (g)	0.91	1.82	2.73	3.66
Polyunsaturated Fatty Acids (PUFA) 18:2 (g)	0.835	1.67	2.5	3.36
PUFA 18:3 (g)	0.062	0.125	0.188	0.223
Cholesterol (mg)	0	0	0	0

**A**: 1 NLEA serving (50 g); **B**: Amount (100 g); **C**: 1 cup cubes (150 g); D: 1 avocado (201 g)

## Compounds of interest in* Persea americana* Mill. cv. Hass

Persea americana Mill cv. Hass avocados are increasingly recognized as a valuable food source of important bioactive compounds (Table 1). Avocados contain unsaturated fatty acids, including the essential oleic acid (*cis*-octadec-9-enoic acid), which constitutes approximately 60–70% of their total fat content and has positive effects on heart health. They also contain significant amounts of carotenoids, such as lutein and zeaxanthin, both of which are important for eye health [37].

**Fig. 4:**
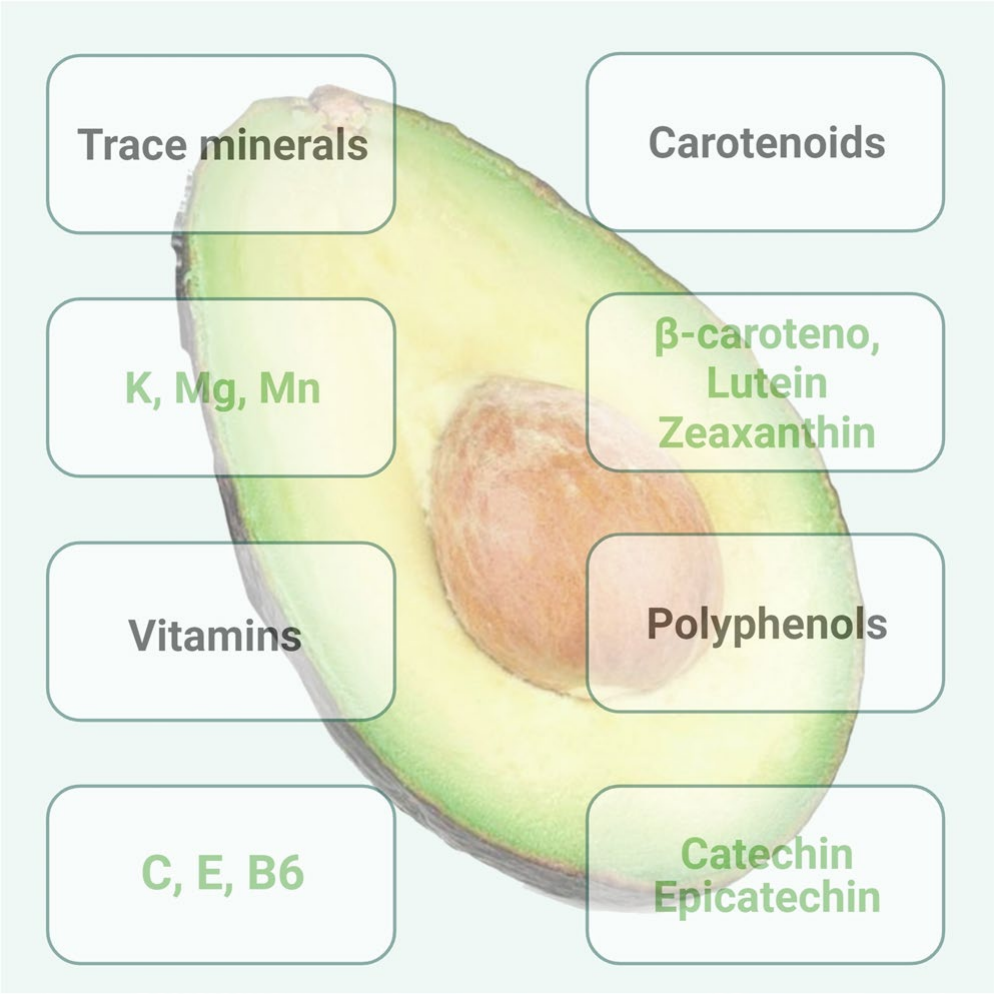
Antioxidant and anti-inflammatory key compounds identified in avocado with potential therapeutic relevance

These carotenoids act as antioxidants and may reduce the risk of age-related macular degeneration (AMD) [38]. The phytosterol and phytostanol content of avocados, particularly beta-sitosterol, can contribute to a decrease in serum cholesterol levels due to their similar chemical structures. Vitamin E, a fat-soluble antioxidant, is abundant in avocado as half avocado provides 1.5 mg or only 10% vitamin E for adults and helps prevent cell damage (Fig. 4) [39]. In addition, polyphenolic compounds, such as catechins and epicatechins, which may exhibit anti-inflammatory and cardioprotective properties, have been identified in avocado [40]. Regular consumption of avocado and these bioactive molecules may have a positive impact on health. Table 2 lists the principal molecules of avocado with antioxidant effects.

**Table 2:** Bioactive compounds from different avocado tissues, their extraction sources, biological effects, and compound types

Compound name	Extract of different parts used	Effect	Type
Persenone	Pulp	Nitric oxide and superoxide generation	Fatty Acid
Gallic acid	Pulp oil and varied by ripening and peeling	Antioxidant activity	Phenolic
Catechin	Seed	Antioxidant and neuroprotective activity	Phenolic
Epicatechin	Avocado fruit pericarp unripe	Antioxidant and neuroprotective activity	Flavonoid
Tocopherol	Pulp	Antioxidant activity	Phenolic
Chlorogenic acid	All fruit	Antioxidant	Phenolic
Scopoletin	All fruit	Antioxidant activity	Phenolic
Lutein (carotene)	Pulp and pulp oil varied by ripening	Antioxidant	Carotenoid
Glutathione	All fruit	Antioxidant activity	Miscellaneous

## Antioxidant Modulation by Molecules Contained in Avocado in Ischemia‒Reperfusion Injury

Antioxidant modulation represents a promising therapeutic strategy for mitigating ischemia–reperfusion injury [41]. In this context, Hass avocados have been the most extensively studied variety because of their popularity and availability [42].

Key antioxidants present in avocados include tannins, carotenoids, *α*, *β*, *γ*, and *δ*-tocopherols, acetogenins, and monounsaturated and polyunsaturated fatty acids. Furthermore, extracts from various parts of the avocado plant contain significant quantities of phenolic molecules with antioxidant capacity. Notably, leaf, peel, and seed extracts demonstrated greater antioxidant capacity than the pulp. A significant positive correlation between the phenolic compound content and antioxidant capacity of avocado extracts has been reported [43]. A study has shown that avocado seeds and peels are rich in polyphenolic compounds, containing levels approximately five to ten times higher than those found in the flesh, thus making them an excellent natural source of phenolic substances. The main antioxidant molecules present in avocado seeds and peels are chlorogenic acid, quinic acid, succinic acid, pantothenic acid, abscisic acid, ferulic acid, gallic acid, sinapic acid, p-coumaric acid, gentisic acid, protocatechuic acid, 4-hydroxybenzoic and benzoic acids, quercetin, quercetin-3-glucoside, quercetin-3-rhamnoside, vanillin, p-coumaroyl-D-glucose, catechins, (-)-epicatechin, and procyanidins. Phenolic compounds in avocado have been shown to reduce oxidation, inflammation, and platelet aggregation in various experimental models, cell cultures, and clinical scenarios [44].

Studies have indicated that these phenolic compounds may mitigate oxidative stress by neutralizing free radicals, consequently reducing inflammation and preventing platelet aggregation, which is crucial for cardiovascular health. The diverse array of antioxidants in avocados showcases their potential for promoting a healthy cellular environment and addressing oxidative stress-related conditions. A recent study assessed the antioxidant capacity of native Mexican avocado seed oil extracted using hexane and its methanolic fraction through ABTS, DPPH, and total antioxidant capacity assays. The seed oil exhibited high radical scavenging activity, approximately 90–91%, while the methanolic fraction showed even greater activity, achieving 99.7% inhibition in the ABTS assay, with an IC₅₀ of 0.035 mg/mL [45]. In another study, phenolic extracts from the peel, pulp, and seed of Persea americana (cv. Hass and Fuerte) were assessed for their antioxidant activity using DPPH, ABTS, and CUPRAC assays and subsequently tested in a refrigerated porcine patty model. The peel and seed extracts demonstrated the highest total phenolic content and strongest radical-scavenging capacity. When incorporated into pork patties, these extracts significantly reduced lipid oxidation (up to ~ 90% inhibition of TBARS) and mitigated protein oxidation during storage [46]. Additionally, several other molecules found in the seeds, peel, and pulp of avocados have beneficial effects on IRI. Some nutraceuticals, such as persenone, help alleviate cerebral damage caused by IRI [47]. Gallic acid, a trihydroxybenzoic acid, is a phenolic molecule found in various fruits, vegetables, and nuts, including avocado pulp, peel, and seed extracts. It scavenges reactive oxygen species and inhibits the production of proinflammatory cytokines, suggesting a potential role in reducing oxidative stress and inflammation [47, 48].

In a study using an isolated rat heart model, administering gallic acid at a dose of 30 mg/kg daily through gavage for 10 days before ischemia via perfusion led to a reduction in cardiac oxidative stress and enhanced functional outcomes. This indicates a partial role in pathways related to oxidative stress. In another study involving a hepatic ischemia and reperfusion injury model, administering gallic acid at doses of 50 and 100 mg/kg body weight before the injury occurred was found to decrease oxidative stress in the liver, significantly reduce alanine aminotransferase and aspartate aminotransferase levels, and improve the activities of catalase and glutathione peroxidase [49–51].

In a rat model of cerebral ischemia-reperfusion induced by bilateral common carotid artery occlusion using the modified Pulsinelli method, pretreatment with *α*-tocopherol (100 mg/kg, administered subcutaneously for 7 days before ischemia) significantly mitigated brain injury. The treated animals exhibited reduced infarct volumes and improved neurological scores compared to untreated controls. Additionally, α-tocopherol enhanced antioxidant defenses by increasing SOD and GSH-Px activities, decreased pro-inflammatory cytokines such as IL-1β, IL-6, and TNF-α, and reduced neuronal apoptosis. These findings suggest that its neuroprotective effects are mediated through antioxidant, anti-inflammatory, and anti-apoptotic mechanisms [52].

α-Tocopherol pretreatment has demonstrated significant neuroprotective effects in experimental stroke models. In animal studies, typical dosages of 100–200 mg/kg of *α*-tocopherol have been shown to reduce cerebral infarct volume by 30–40%, improve neurological deficit scores, and enhance long-term cognitive outcomes. These protective effects are achieved through the inhibition of lipid peroxidation, preservation of blood-brain barrier integrity, and reduction of neuroinflammation. α-Tocopherol (30 mg/kg, i.p.) has shown beneficial effects in experimental renal ischemia, including reduction in tubular damage scores and cast formation, decreased blood urea nitrogen (BUN) and creatinine elevations, protection against oxidative DNA damage in renal tubular cells, and preservation of glomerular filtration capacity [53].

In a myocardial ischemia model, α-tocopherol demonstrated cardioprotective properties by reducing neutrophil and monocyte infiltration into damaged heart tissue, decreasing the production of reactive oxygen species and peroxidized lipids, and mitigating cardiac ischemia-reperfusion injury while preserving cardiac function in male C57BL/6 mice [54].

Lutein, the primary carotenoid found in avocados, has demonstrated promising protective properties in experimental studies of retinal ischemia. Administration of lutein at doses of 10–20 mg/kg prior to a retinal ischemic event significantly decreased oxidative stress indicators, including malondialdehyde and 4-hydroxynonenal, while preserving retinal neuron viability. These protective mechanisms involve direct antioxidant effects on retinal ganglion cells, reduction of inflammatory cytokines (IL-1*β* and TNF-*α*), and preservation of photoreceptor function [54].

In a rat model of intestinal IRI induced by transient occlusion of the superior mesenteric artery followed by reperfusion, lutein administration prior to ischemia reduced oxidative tissue damage and preserved intestinal morphology, mainly by attenuating lipid peroxidation [55]. In a sciatic nerve IRI model, oral administration of lutein before ischemia and after reperfusion reduced neuropathic pain and nerve injury by decreasing oxidative stress and suppressing inflammatory mediators, such as NF-*κ*B and TNF-*α*, while preserving endogenous antioxidant defenses [56].

## Anti-Inflammatory Compounds from Avocado reduce Inflammatory Damage in Ischemia‒Reperfusion Injury

Several studies have investigated the anti-inflammatory properties of avocados. Avocado oil contains a significant amount of essential fatty acids, with oleic acid comprising 47.20% of the unsaturated fatty acids. Non-pharmacological agents that modulate the expression of proinflammatory mediators are effective and safe alternatives for treating IRI [45, 57, 58]. Avocado/soybean unsaponifiables (ASU) is one of the most extensively studied avocado-derived pharmaceutical preparations. ASU is a specific extract composed of the unsaponifiable fraction of avocado oil (approximately one-third) combined with soybean oil (approximately two-thirds).

Studies have demonstrated that ASU reduces oxidative stress markers and inflammatory cytokines in conditions that share pathophysiological mechanisms with IRI. ASU is commonly administered at a dosage of 300 mg/day for three months to treat patients with osteoarthritis. It has been demonstrated to reduce pro-inflammatory mediators, including IL-1*β*, TNF-*α*, prostaglandin E2, and matrix metalloproteinases, partially through the modulation of NF-κB signaling. These mediators play critical roles in ischemia-reperfusion injury, and ASU is associated with a favorable safety profile [59, 60].

Oral administration of 600 mg/kg/day ASU for 10 days significantly mitigated brain damage induced by ischemia-reperfusion. ASU treatment reduced lipid peroxidation and neuronal apoptosis, enhanced endogenous antioxidant defenses, including superoxide dismutase and catalase, and suppressed inflammatory responses, as evidenced by decreased levels of tumor necrosis factor-alpha in the hippocampus and prefrontal cortex [61, 62].

Chlorogenic acids, which have anti-inflammatory effects, are present throughout avocado fruit, especially in the peel and seed. They have demonstrated neuroprotective effects against damage caused by ischemia–reperfusion injury in rat models [63]. Studies using middle cerebral artery occlusion (MCAO) models in rats have demonstrated that chlorogenic acid pretreatment (10–50 mg/kg) significantly reduces infarct volume, improves neurological scores, and enhances long-term cognitive recovery [64, 65].

Chlorogenic acid was administered intraperitoneally at doses of 2.5, 5, and 10 mg/kg on two occasions: 10 min before ischemia and 10 min before reperfusion. Chlorogenic acid administration resulted in significant improvements in hepatic function and histological appearance, as well as a reduction in oxidative stress, as evidenced by decreased hepatic lipid peroxidation and altered glutathione levels. Furthermore, chlorogenic acid treatment attenuated the levels of serum tumor necrosis factor-α, inducible nitric oxide synthase, and cyclooxygenase-2 protein and mRNA expression, which were upregulated following ischemia-reperfusion injury [66].

Scopoletin, a phenolic coumarin, is present in various plants, including the leaves, peels, and seeds of avocados. Scopoletin has demonstrated cardioprotective effects in a rat model of isoproterenol-induced myocardial infarction, where it was administered as a preventive measure before cardiac injury (50-mg dosage of scopoletin for 28 days). It mitigated myocardial damage by reducing oxidative stress, inflammation, and apoptosis, as evidenced by improved histopathology and the modulation of biochemical markers of injury [67]. Although these findings suggest the involvement of antioxidant and cytoprotective mechanisms, the evidence remains largely correlative, as the specific molecular targets and signaling pathways directly regulated by scopoletin have not been clearly defined. In ischemia-reperfusion injury models, particularly those involving heart tissue, scopoletin enhances the activity of antioxidant enzymes while reducing inflammation and tissue damage [68].

The antioxidant and anti-inflammatory properties of avocados are largely attributed to their monounsaturated fatty acids (MUFAs) and polyphenolic compounds. Oleic and palmitic acids are the most abundant fatty acids in native Mexican fruits. The pulp of Hass avocados has an oleic acid content of approximately 66.0–71.1% and palmitic acid content of 14.69–22.64% [69]. Oleic acid, the primary MUFA, is linked to decreased lipid peroxidation, enhanced membrane stability, and the reduction of redox-sensitive inflammatory pathways during oxidative stress, a phenomenon pertinent to ischemia–reperfusion injury. Oleic acid exerts direct and indirect suppression of NF-κB, a key inflammatory transcription factor, and reduces NLRP3 inflammasome activation, thereby limiting production of pro-inflammatory cytokines IL-1β and IL-18 [70].

Although direct studies on avocado oil in myocardial ischemia-reperfusion models are limited, dietary supplementation with high-oleic acid sources, including avocado, has been associated with reduced myocardial infarct size in experimental models, improved post-ischemic contractile recovery, and enhanced survival of cardiomyocytes under hypoxic conditions [71, 72].

The hallmark of ferroptosis is the oxidation of polyunsaturated fatty acids (PUFAs). Hass avocados are exceptionally rich in monounsaturated fatty acids (MUFAs), particularly oleic acid. Research has shown that increasing the ratio of monounsaturated fatty acids (MUFAs) to polyunsaturated fatty acids (PUFAs) in cell membranes can render them less susceptible to lipid peroxidation, effectively acting as an endogenous inhibitor of ferroptosis. Furthermore, avocado-derived acetogenins and tocopherols (vitamin E) act as potent radical-trapping antioxidants (RTAs) that can terminate the autoxidative chain reaction of lipid membranes [73].

### Future Directions and Perspectives

An essential aspect to consider when assessing the therapeutic potential of compounds derived from avocados is that the consumption of whole avocados offers a complex array of bioactive constituents that may exert synergistic protective effects that are not evident when the components are isolated. The distinctive composition of Hass avocado facilitates potential synergistic interactions between water-soluble polyphenols, such as chlorogenic acid and gallic acid, and lipid-soluble antioxidants, including tocopherols and carotenoids, collectively providing comprehensive cellular protection against oxidative damage [45, 74]. Furthermore, the natural lipid composition of avocados aids in the absorption of fat-soluble vitamins and carotenoids in the intestines, potentially leading to higher bioavailability than when these nutrients are taken as isolated supplements. While there are few direct comparative studies, new evidence indicates that whole avocado extracts may offer greater protective benefits than isolated compounds at equivalent concentrations [75]. Although the whole-food matrix offers nutritional benefits, it also presents translational challenges. A significant limitation in the literature is the absence of physiologically relevant dose–response data. Some experimental studies utilize extract concentrations or purified compounds at levels unlikely to be achieved through dietary consumption, complicating the assessment of whether the observed antioxidant and anti-inflammatory effects are nutritionally. Furthermore, there is considerable heterogeneity in the types of avocado-derived preparations studied, including pulp, oil, peel, seed extracts, and unsaponifiable fractions, often with incomplete phytochemical characterization. This variability hinders reproducibility, cross-study comparability, and the standardization of potential clinical formulations. Different routes of administration have demonstrated varying efficacy in delivering avocado-derived compounds. Oral supplementation remains the most clinically viable strategy; however, it encounters significant bioavailability challenges that may limit therapeutic effectiveness [74, 76]. Polyphenols and carotenoids undergo significant changes in the stomach and liver. They are also modified by gut bacteria. The forms that circulate in the body can differ significantly from the original compounds in terms of structure and function. To date, few studies have examined how these altered forms move through the body or accumulate in organs sensitive to low blood flow. This is important in cases of ischemia–reperfusion injury (IRI), where the timing, extent of tissue penetration, and concentration are crucial for effectiveness. However, no randomized controlled trials have specifically tested Hass avocados or their active compounds in preventing or reducing ischemia–reperfusion injury in humans [71].

Nonetheless, no randomized controlled trials have specifically investigated the efficacy of Hass avocados or their active compounds in the prevention or mitigation of ischemia–reperfusion injury in humans. To date, no clinical studies have specifically examined intervreperfusion injurnullentions derived from Hass avocados in relation to ischemia-reperfusion injury (IRI). Existing human research has predominantly focused on broader cardiovascular or metabolic outcomes. These studies encompass improvements in autonomic and cardiovascular recovery following exercise in healthy individuals, as well as dietary interventions involving avocados in patients with a history of ischemic stroke. The primary focus of these studies is on lipid profiles and secondary prevention outcomes, rather than [77] mechanistic endpoints associated with reperfusion injury.

Most preclinical studies use laboratory systems or animal models that do not fully mimic the complex nature of ischemia–reperfusion injury. These models fail to capture key aspects, such as mitochondrial problems, blood vessel activation, calcium overload, and immune responses. Therefore, although the data from these studies are promising, applying them directly to human IRI cases is premature. Initial clinical trials using Hass avocados for IRI should begin by assessing safety, feasibility, and biomarker responses. Suitable participants could be those facing planned ischemic events, such as heart surgery, organ transplants, or procedures to restore blood flow. Interventions could include avocado-based products rich in phenolic compounds, carotenoids, or healthy fats, or controlled whole-food preparations [78]. Importantly, the dosage should be based on what people can realistically eat and absorb, not on drug-like assumptions. Key outcomes should include proven biomarkers of oxidative stress, inflammation, blood vessel problems, and organ damage related to IRI Despite encouraging preclinical results, significant knowledge gaps remain concerning bioavailability, tissue targeting, dose relevance, and molecular mechanisms of action under ischemia–reperfusion injury (IRI) conditions. In this context, Hass avocado and its bioactive components should be regarded as complementary elements of dietary strategies rather than as standalone pharmacological interventions, in alignment with the translational framework of plant-based nutrition research [79, 80]. This review highlights the potential of bioactive compounds in Hass avocados to modulate oxidative stress and inflammation associated with ischemia–reperfusion injury. However, rigorous in vivo studies, standardized formulations, nutritionally relevant dosing, and well-designed controlled clinical trials are essential to substantiate their translational applicability. Future studies should prioritize organ systems with the highest translational importance, such as the cardiovascular, renal, intestinal, and cerebral systems. Emphasis should be placed on well-controlled in vivo experiments and standardized avocado-derived formulations to better understand their bioavailability, dose–response relationships, and synergistic effects. Ultimately, controlled clinical trials will be crucial for converting current experimental findings into strategies applicable in clinical settings.

## Conclusions

In summary, this comprehensive review details the molecular mechanisms underlying ischemia–reperfusion injury and discusses the potential role of bioactive compounds found in Hass avocados in modulating oxidative stress and inflammation associated with this condition. The fruit’s composition–including monounsaturated fatty acids, antioxidants such as tocopherols and carotenoids, and other phytochemicals–may contribute to these biological effects through complementary mechanisms. Current evidence indicates that bioactive compounds in Hass avocados demonstrate antioxidant, anti-inflammatory, and cytoprotective properties in experimental models. Nevertheless, although these findings are encouraging, their applicability to clinical ischemia-reperfusion injury outcomes in humans has yet to be definitively established. Future research should focus on elucidating specific molecular interactions, assessing bioavailability under physiological conditions, and exploring potential synergistic effects with established therapeutic strategies. Such studies may help clarify whether avocado-derived bioactives can serve as adjuncts, rather than standalone, approaches for the management of ischemia–reperfusion injury.

Although preclinical studies provide mechanistic insights, the clinical application of Hass avocado bioactives in ischemia–reperfusion contexts remains limited. Experimental doses often exceed levels achievable through habitual dietary intake, and bioavailability may vary depending on the food matrix, processing, and formulation. Moreover, most current evidence derives from in vitro and animal models, with a scarcity of well-designed human studies evaluating clinically relevant ischemia–reperfusion injury endpoints. Addressing these limitations through standardized formulations, dose-optimization studies, and rigorously designed clinical trials will be essential before drawing definitive conclusions regarding their therapeutic applicability.

## Supplementary Information

Below is the link to the electronic supplementary material.

Supplementary Material 1 (DOCX 67.9 KB)

Supplementary Material 2 (DOCX 15.1 KB)

## Data Availability

No datasets were generated or analysed during the current study.

## Abbreviations

IRI: Ischemia-reperfusion injury
ROS: Reactive Oxygen Species
RNS: Reactive Nitrogen Species
BBB: Blood-Brain Barrier
HIFs: Hypoxia-Inducible Factors
PHD: Prolyl Hydroxylase
NRF2: Nuclear Factor Erythroid 2–Related Factor 2
NF-kB: Nuclear Factor kappa-light-chain-enhancer of activated B cells
MAPK: Mitogen-Activated Protein Kinase
VEGF: Vascular Endothelial Growth Factor
VEGF-R1: Vascular Endothelial Growth Factor Receptor 1
VEGF-R2: Vascular Endothelial Growth Factor Receptor 2
MMPs: Matrix Metalloproteinases
SOD: Superoxide Dismutase
CAT: Catalase
GPX: Glutathione Peroxidase
DNA: Deoxyribonucleic Acid
NO: Nitric Oxide
ATP: Adenosine Triphosphate
ETC: Electron Transport Chain
mPTP: Mitochondrial Permeability Transition Pore
GPX4: Glutathione Peroxidase 4
NOS: Nitric Oxide Synthase
ASU: Avocado Soybean Unsaponifiable
MUFAs: Monounsaturated Fatty Acids
PUFAs: Polyunsaturated Fatty Acids
MF: Monounsaturated Fatty acid
