# Supplementary material for: Hass Avocado Bioactive Compounds Attenuating Oxidative Stress and Inflammation in Ischemia–reperfusion Injury: An Integrative Review
Source: Plant Foods Hum Nutr. 2026 May 8;81(2):52. doi: 10.1007/s11130-026-01482-4 (PMC13156230; doi:10.1007/s11130-026-01482-4)
Supplement: Supplementary file 1 — Supplementary Material 1 (DOCX 67.9 KB) [file 11130_2026_1482_MOESM1_ESM.docx]

**Table 3**. Effects of Hass avocado derived bioactive compounds on oxidative stress and inflammation in ischemia–reperfusion models

| Bioactive compound avocado | Subject | Study type | Dose | Main effects | Reference |
| --- | --- | --- | --- | --- | --- |
| Lutein | C57BL/6N male mice | In vivo | 0.2 mg/kg | ↓ number of apoptotic cells, cell loss  ↓ NT and nuclear PAR immunoreactivity | [82] |
|  | Male sprague-Dawley | In vivo | 0.5 mg/kg | MDA↓, GSH↑, NO ↑, MPO ↓  Necrosis, edema and PNML infiltration ↓  Collagen, fibroblast proliferation and vascular density↑  Epidermal thickness ↑ | [83] |
|  | Cardiac microvascular endothelial cells  Male Wistar rats | In vitro and in vivo | 20 μM  20 mg/kg | ↓ferroptosis in cardiomyocytes  ↑MDM2 | [84] |
|  | Male mice | In vivo | 25 mg/kg/day | ↓ ROS, MDA  ↓TNF-α, IL-1β e IL-6  ↓NLRP3  ↑PPAR-γ | [85] |
| Scopoletin | Albino male rats | In vivo | 25 and 50 mg/kg | ↓ cTnT, CK-MB and LDH  ↓ MDA, Caspase 3, Caspase 9  ↑Catalase and SOD | [86] |
| Soybean unsaponificable (ASU) | Male Sprague-Dawley rats | In vivo | 600 mg/kg/day | ↓ MDA, TNF-α, number of apoptotic neurons in the prefrontal cortex  ↑SOD | [87] |
| Gallic acid | Adult Spraque–Dawley albino rats | In vivo | 50 and 100 mg/kg | ↓ALT, AST, LDH activities and MDA levels  ↑CAT and GPx | [88] |
| Epicatechin | H9C2 cells  Adult male Sprague-Dawley rats | In vitro and in vivo | 100 uL  Concentrations (1, 2.5, 5, 10, 20, 40, 100 µM)  1 mg/kg/day  2 mg/kg/day | ↓ autophagy and ferroptosis  ↓ levels of lipid ROS in cells  ↓ROS and Fe^2+^, ferroptosis in vivo | [89] |
| Chlorogenic acid | Male Sprague-Dawley rats  L02 cells | In vitro and in vivo | 100 mg/kg.day  Concentrations  (12.5, 50, or 200 µM) | ↓ALT, AST, MDA, TNF-α, and IL-1β levels  ↓ apoptosis  Improve SOD and GSH levels | [90] |

NT: nitrotyrosine; PAR: poly(ADP-ribose); MDA: malondialdehyde; GSH: glutathione; NO: nitric oxid ; MPO: myeloperoxidase; PNML: polymorphonuclear leucocyte; cTnT: Cardiac troponin T, CK-MB: Creatine Kinase-MB; LDH: lactate dehydrogenase; MDM2: mouse double minute-2
