# Supplementary material for: Hass Avocado Bioactive Compounds Attenuating Oxidative Stress and Inflammation in Ischemia–reperfusion Injury: An Integrative Review
Source: Plant Foods Hum Nutr. 2026 May 8;81(2):52. doi: 10.1007/s11130-026-01482-4 (PMC13156230; doi:10.1007/s11130-026-01482-4)
Supplement: Supplementary file 2 — Supplementary Material 2 (DOCX 15.1 KB) [file 11130_2026_1482_MOESM2_ESM.docx]

**Supplementary Material**

81. Vázquez-Galán YI, Guzmán-Silahua S, Trujillo-Rangel WÁ, Rodríguez-Lara SQ (2025) Role of Ischemia/Reperfusion and Oxidative Stress in Shock State. Cells 2025, Vol 14, 14:. <https://doi.org/10.3390/cells14110808>

82. Li SY, Fu ZJ, Ma H, Jang WC, So KF, Wong D, et al. Effect of Lutein on Retinal Neurons and Oxidative Stress in a Model of Acute Retinal Ischemia/Reperfusion. Invest Ophthalmol Vis Sci. The Association for Research in Vision and Ophthalmology; 2009;50:836–43. https://doi.org/10.1167/IOVS.08-2310

83. Akdemir O, Eyuboglu A, Cetin EO, Uyanikgil Y. Lutein Protects Ischemic Skin Flaps via Antioxidant and Anti-Inflammatory Mechanisms in a Rat Model of Ischemia-Reperfusion Injury. J Invest Surg [Internet]. J Invest Surg; 2025 [cited 2026 Jan 25];38. https://doi.org/10.1080/08941939.2025.2528341

84. Shi P, Sha Y, Wang X, Yang T, Wu J, Zhou J, et al. Targeted Delivery and ROS-Responsive Release of Lutein Nanoassemblies Inhibit Myocardial Ischemia-Reperfusion Injury by Improving Mitochondrial Function. Int J Nanomedicine [Internet]. Int J Nanomedicine; 2024 [cited 2026 Jan 30];19:11973–96. https://doi.org/10.2147/IJN.S488532

85. Nie C, Liu Z, Zhang L, Liu C, Jiang H, Liu M. Lutein Alleviate Acute Lung Injury Induced by Limb Ischemia-Reperfusion Through PPAR-γ/PI3K/AKT/NLRP3 Signaling. Mediators Inflamm [Internet]. John Wiley & Sons, Ltd; 2025 [cited 2026 Jan 30];2025:2371545. https://doi.org/10.1155/MI/2371545

86. Rong N, Yang R, Ibrahim IAA, Zhang W. Cardioprotective Role of Scopoletin on Isoproterenol-Induced Myocardial Infarction in Rats. Applied Biochemistry and Biotechnology 2022 195:2 [Internet]. Springer; 2022 [cited 2026 Jan 25];195:919–32. https://doi.org/10.1007/S12010-022-04123-Z

87. Eser O, Songur A, Yaman M, Cosar M, Fidan H, Sahin O, et al. The protective effect of avocado soybean unsaponifilables on brain ischemia/reperfusion injury in rat prefrontal cortex. Br J Neurosurg. 2010;25:701–6. https://doi.org/10.3109/02688697.2010.520767

88. Bayramoglu G, Kurt H, Bayramoglu A, Gunes HV, Degirmenci İ, Colak S. Preventive role of gallic acid on hepatic ischemia and reperfusion injury in rats. Cytotechnology [Internet]. Cytotechnology; 2015 [cited 2026 Jan 25];67:845–9. https://doi.org/10.1007/S10616-014-9724-1

89. Junhong K, Yun T, Guangxing S, Yuhan D, Qian X, Haowen Z. (-)-Epicatechin protects against myocardial ischemia/reperfusion injury via autophagy-dependent ferroptosis. Aging [Internet]. Aging (Albany NY); 2024 [cited 2026 Jan 27];16:2181–93. https://doi.org/10.18632/AGING.205477

90. Li K, Feng Z, Wang L, Ma X, Wang L, Liu K, et al. Chlorogenic Acid Alleviates Hepatic Ischemia-Reperfusion Injury by Inhibiting Oxidative Stress, Inflammation, and Mitochondria-Mediated Apoptosis In Vivo and In Vitro. Inflammation [Internet]. Inflammation; 2023 [cited 2026 Jan 29];46:1061–76. https://doi.org/10.1007/S10753-023-01792-8
